# Preliminary Preclinical Evaluation of Innovative Bone Scaffolds Composed of Natural Sources–Whey Protein Isolate and Pearl Powder

**DOI:** 10.3390/ijms26167939

**Published:** 2025-08-17

**Authors:** Daniel K. Baines, Jaroslaw Rachuna, Aleksandra Hnydka, Agnieszka Michalak, Timothy E. L. Douglas, Katarzyna Klimek

**Affiliations:** 1 School of Engineering, Lancaster University, Gillow Avenue, Lancaster LA1 4YW, UK; d.baines3@lancaster.ac.uk (D.K.B.); t.douglas@lancaster.ac.uk (T.E.L.D.); 2 Chair and Department of Biochemistry and Biotechnology, Medical University of Lublin, Chodzki 1 Street, 20-093 Lublin, Poland; jaroslaw.rachuna@umlub.pl (J.R.); d.aleksandrahnydka@umlub.edu.pl (A.H.); 3 Independent Laboratory of Behavioral Studies, Medical University of Lublin, Chodzki 4a Street, 20-093 Lublin, Poland; agnieszka.michalak@umlub.pl

**Keywords:** whey protein isolate, pearl powder, bone, scaffold, cell-biomaterial interactions, *Danio rerio*

## Abstract

The aim of this work was to produce bone scaffolds containing whey protein isolate and pearl powder and to conduct a preliminary assessment of the biomedical potential in vitro and in vivo. This included analysis of structural, physicochemical, mechanical, and biological properties, which revealed that biomaterials containing pearl powder exhibited an enhanced porous structure, increasing absorptive properties, and decreasing proteolytic capacity with increasing inorganic component content. Pearl powder content in the biomaterials did not clearly influence their mechanical properties or their ability to release calcium ions, as well as proteins. Extracts obtained from all tested biomaterials showed no cytotoxicity in vitro. The surfaces of all biomaterials promoted normal human osteoblast growth, proliferation, and osteogenic differentiation. Furthermore, all biomaterials did not display toxicity in vivo, but no changes in Danio rerio were observed after evaluation of the biomaterial containing the highest amount of pearl powder–10% *v*/*w* (marked as WPI/P10). Taking all the obtained results into account, it appears that this biomaterial can be promising for bone scaffolds and similar applications, thanks to its porous structure, high cytocompatibility in vitro, and lack of toxicity in vivo. However, advanced studies will be conducted in the future.

## 1. Introduction

Although bone is a dynamic tissue and has a significant capacity for self-regeneration, bone defects remain one of the most serious challenges facing modern medicine. Bone is the second most frequently transplanted tissue in the human body, more often in men than in women, and also more frequently in people over the age of 50. The cost of treating bone fractures in the United States is projected to reach USD 25 billion. Large defects, known as critical-sized defects (CSDs), ranging in size from 2 cm to over 12 cm (depending on the location in the region of the skeleton), require the use of a bone graft, as they cannot regenerate naturally. According to estimates, over 2.2 million bone grafts are used worldwide each year. Unfortunately, the numerous limitations associated with the use of bone grafts (autografts, allografts, or xenografts) are increasingly leading orthopedic surgeons to turn to tissue engineering products (TEPs). In the case of bone biomaterials, composite or hybrid scaffolds are the most popular; these contain natural or synthetic polymers (especially collagen, gelatin, and polylactic acid) and ceramics (particularly hydroxyapatite–HAp, tricalcium phosphate–TCP, tetracalcium phosphate–TTCP, and amorphous calcium phosphate–ACP) [1,2,3,4,5,6,7,8,9,10,11].

To date, many biomaterials have been developed that show promising features as bone scaffolds. However, none of them meet all the requirements, such as biomimicry, appropriate porosity, pore size, mechanical strength, and osteoinductive and osteoconductive properties. Therefore, new prototypes are continually being developed that will demonstrate improved properties compared to existing ones. Alternative fabrication methods, as well as polymer and ceramic components, are particularly sought after [6,12,13,14,15,16,17,18].

One interesting alternative material to the ceramic is pearl powder. Pearl powder is a natural biomineral found in bivalves. It consists of 95% inorganic phase, primarily calcium carbonate – CaCO_3_ (aragonite), and 5% organic phase, primarily proteins, glycoproteins, and polysaccharides. It has even been suggested that pearl powder has greater osteoconductive properties than HAp, thanks to the ability to release bone morphogenetic proteins (BMPs) [19,20,21]. In addition to its osteogenic properties, pearl powder also possesses antioxidant, anti-inflammatory, and anti-apoptotic activity [22,23]. Combining pearl powder with a polymer matrix allows for the production of a composite or hybrid biomaterial with high cytocompatibility in vitro and biocompatibility in vivo [24,25,26,27]. For example, Guo et al. [28] developed a 3D-printed scaffold composed of polylactic acid (PLA) and pearl powder and demonstrated that the addition of this biomineral increased in vitro bioactivity and the ability of stem cells to proliferate and undergo osteogenic differentiation. Yang et al. [29] developed a 3D-printed hybrid bone scaffold containing pearl powder and gelatin methacrylate (GelMa) enriched with vascular endothelial growth factor (VEGF). The authors demonstrated that the biomaterial possesses high cytocompatibility in vitro, supporting the adhesion and osteogenic differentiation of stem cells. Furthermore, in vivo studies demonstrated that this scaffold, thanks to its controlled ability to release VEGF, supports angiogenesis. Zhang et al. [30], in turn, fabricated composites composed of pearl powder and polycaprolactone and showed that increasing pearl powder enhanced mechanical properties and bioactivity, as well as cell adhesion, proliferation, and differentiation. These reports clearly indicate that pearl powder is a promising ingredient in bone biomaterials. Whilst similar materials such as shell nacre offer potential in tissue regeneration when compared to similar materials such as shell nacre, pearl powder offers the advantage of a uniformly smaller particle size. This finer size enables better dispersion within the hydrogel, resulting in a more homogeneous polymer–pearl powder mixture and a correspondingly more uniform hydrogel. In contrast, shell nacre particles are generally coarser, which can lead to an uneven distribution. A more uniform hydrogel not only improves consistency but also allows for greater predictability in its mechanical and biological behavior. Furthermore, the established use of pearl powder in the cosmetic industry ensures wider availability and more consistent quality control [22,31,32].

On the other hand, in the case of polymer components, whey protein isolate (WPI) has recently attracted considerable scientific interest. This protein is also of natural origin, being produced from milk as a byproduct during cheese production. Previously, WPI constituted up to 90% of the waste generated by the cheese industry. Repurposing this abundant by-product for biomedical use, combined with its global availability, represents a significant step toward sustainable production of biomaterials for tissue regeneration. In contrast to other natural polymers such as plant-derived cellulose, starch, or alginates [33]; animal-derived collagen or gelatin [34]; or marine-derived materials like chitosan from shellfish [35], the use of WPI offers the unique advantage of being sourced without additional cultivation, harvesting, or harm to animals, while simultaneously utilizing an existing waste stream. WPI is rich in β-lactoglubulin, which promotes cell adhesion, proliferation, and differentiation; furthermore, its aqueous solution has the ability to form a mechanically stable hydrogel when heated above 90 °C [36,37,38,39,40,41]. The resulting hydrogel is also characterized by high cytocompatibility. Therefore, several bone scaffolds based on WPI have been developed to date. For example, Dziadek et al. [42] developed bone composites composed of WPI and aragonite. Depending on the inorganic phase content, the biomaterials exhibited varying pore sizes ranging from 18 to 778 µm. The biomaterials also exhibited the ability to degrade over time and promoted the growth and differentiation of osteoblast-like MG-63 cells. Ivory-Cousins et al. [37] fabricated bone composites consisting of WPI and calcium silicate (CaSiO_3_) and demonstrated that the addition of 5% CaSiO_3_ to the biomaterial based on WPI increased the biomaterial’s swelling capacity, mechanical properties, and enhanced the proliferation and differentiation of human osteoblasts.

However, to date, bone scaffolds containing WPI and pearl powder have not been produced. Therefore, the aim of this work is to evaluate the structural, physicochemical, mechanical, and biological properties of new biomaterials composed of 40% *w/v* WPI and 2.5%, 5%, 7.5%, and 10% *w/v* pearl powder. It was hypothesized that composites containing WPI and pearl powder would be characterized by high cytocompatibility in vitro and lack of toxicity in vivo, due to the combination of the properties of both components. Furthermore, it was hypothesized that the addition of pearl powder would increase the absorptive properties of the composites and their mechanical properties. To verify these hypotheses, a series of analyses was conducted using the latest research techniques.

## 2. Results and Discussion

### 2.1. Microstructure and Macrostructure of Biomaterials

**Figure 1:**
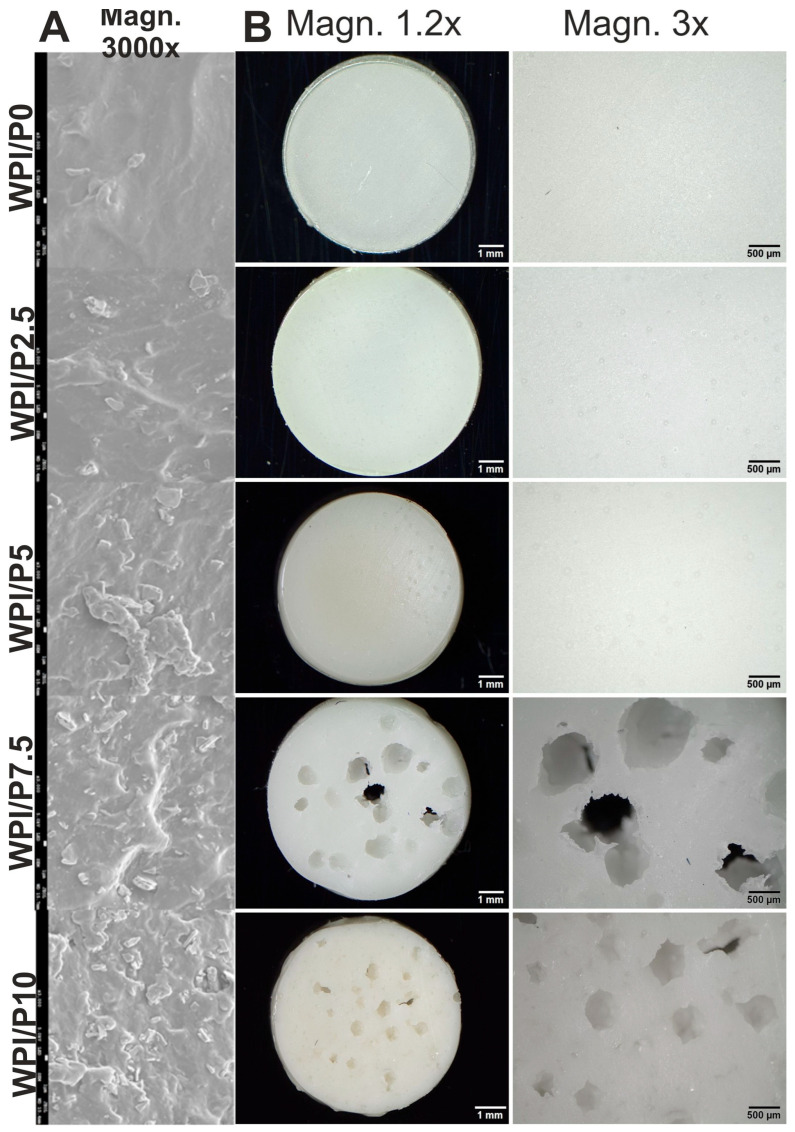
SEM images presenting microstructure of stiff but flexible hydrogels, Magn. 3000×, scale bar = 1 μm (**A**) and stereoscopic microscope images presenting macrostructure of stiff but flexible hydrogels (**B**), Magn. 1.2.×, scale bar = 1 mm and Magn. 3×, scale bar = 500 μm.

Scanning electron microscopy (SEM) analysis (Figure 1A) revealed that the number of visible precipitates on the surfaces of biomaterials increased with increasing percentage of pearl powder. Furthermore, stereoscopic microscope observations (Figure 1B) showed that the surface of the biomaterial without pearl powder (WPI/P0) and the surfaces of biomaterials containing small amounts of such powder (WPI/P2.5 and WPI/P5) did not have visible pores. However, in the case of the biomaterials with the highest pearl powder content (WPI/P7.5 and WPI/P10), visible pores were observed, giving the biomaterials a morphology reminiscent of “Swiss cheese”. The reason for this phenomenon is unclear, but it may be related to the heating of pearl powder. Heating pearl powder, which primarily contains CaCO_3_, can lead to the decomposition of CaCO_3_ and the release of carbon dioxide (CO_2_) gas in a process called calcinations [43]. It is well known that CO_2_ is considered one of the most popular porogens used in biomaterials engineering [44,45]. Therefore, in WPI/P7.5 and WPI/P10 biomaterials, the amount of CaCO_3_ had to be large enough to create visible pores. The presence of pores is very beneficial for the future fate of bone scaffolds. It has been recommended that bone scaffolds should have pores of at least 100 µm, which are necessary for cell growth and proliferation, while pores above 300 µm are crucial for angiogenesis and the growth of new bone tissue [15,46,47]. Interestingly, in our previous works, Dziadek et al. noticed that pores occurred in WPI hydrogels after the addition of bioactive glass particles [42], while Gupta et al. observed pore formation in WPI hydrogels after the addition of aragonite particles [48].

### 2.2. Fourier-Transform Infrared (FTIR) Analysis of Biomaterials

**Figure 2:**
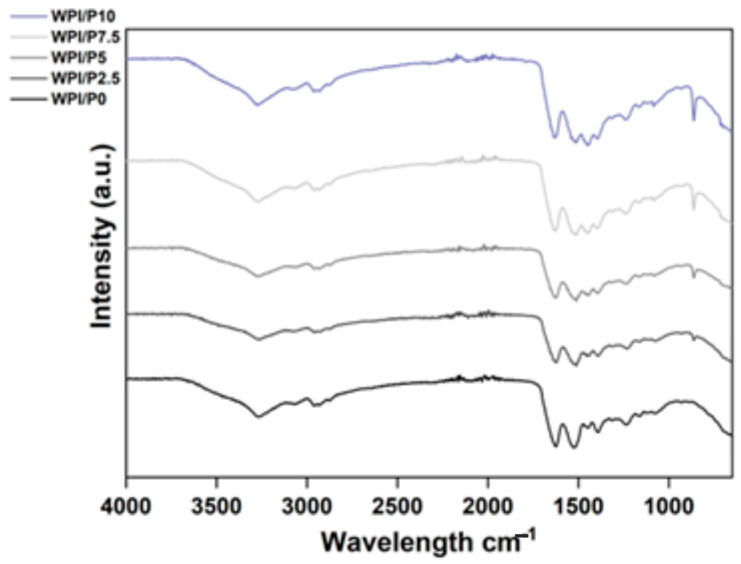
FTIR analysis of the WPI/Pearl hydrogels (intensity in arbitrary units (a.u.) with wavenumber). The samples increase in pearl concentration in an ascending order, with the WPI/P0 control, with no pearl powder (black) at the bottom.

FTIR analysis was conducted to confirm the successful incorporation of the pearl powder into the WPI hydrogel. The results, observable in Figure 2, demonstrated peaks at bands 3271 cm^−1^, 2955 cm^−1^, 1615 cm^−1^, 1520 cm^−1^, 1444 cm^−1^, 1399 cm^−1^, 1233 cm^−1^, and 859 cm^−1^. Further analysis of the interactions in each region can be observed in Table 1.

The results of the FTIR analysis are consistent with the study of Li et al. involving pearl powder [23] as a biomaterial component. One observation was an increase in intensity in the 858 cm^−1^ region; this region has previously been associated with the carbonate bending vibration in aragonite. Previously, the carbonate ion CO_3_^2−^ has demonstrated an increase in intensity in the region of 855–860 cm^−1^. For example, Ni and Ratner [49] suggested a peak for aragonite at 585 cm^−1^. Pearl powder is primarily composed of calcium carbonate (CaCO_3_). The CaCO_3_ is primarily in the form of aragonite crystals, with some calcite. Therefore, an increase in intensity in this region, particularly a linear increase with increasing pearl powder concentration, suggests the successful incorporation of the pearl powder into the WPI hydrogel and, therefore, the successful manufacturing of WPI/Pearl hydrogels. Furthermore, the results suggest an increase in intensity in the 1444 cm^−1^ region. Interestingly, the regions between 1390 and 1470 cm^−1^ have been associated with CO_3_^2−^ asymmetric stretching, providing further evidence of successful pearl powder incorporation.

**Table 1:** The wavenumbers were acquired by FTIR, and the potential underlying interactions. This table has been adapted from Baines et al. [50].

Wavenumber cm^−1^	Region/Potential Interactions
3271	Amide A—O-H and N-H stretching
2955	Unfunctional CH–CH stretching modes, both symmetric and asymmetric
1615	Amide I
	Arginine side chain symmetric stretching vibrations—CN_3_H_5+_
	Asparagine side chain in-plane bending vibrations—NH_2_
	Glutamine side chain in-plane bending vibrations—NH_2_
	Lysine side chain antisymmetric in-plane bending vibrations—NH_3+_
	Tryptophan side chain stretching vibration CC, stretching vibration C = C, NH
	Tyrosine side chain stretching vibrations CC ring, in-plane bending vibrations CH
1520	Amide II
	Tyrosine—OH, CC stretching vibrations, CH in-plane bending vibrations
	Lysine side chain interaction—symmetric in-plane bending vibrations—NH_3+_
	Tryptophan—stretching vibration—CN, in-plane bending vibration CH, NH
	stretching vibrations CC ring, in-plane bending vibrations CH
1444	CO_3_^2−^–Asymmetric stretching
	Proline—CN stretching vibrations, CH_2_ in-plane bending vibrations, CH_3_ antisymmetric bending vibrations
	Glutamic acid side chain interactions—in-plane bending vibrations—CH_2_
	Glutamic acid side chain interactions—in-plane bending vibrations—CH_3_
	Glutamine side chain interactions—in-plane bending vibration—CH_2_
	Histidine side chain interactions—in-plane bending vibrations CH, stretching vibrations CN
	Lysine side chain interactions—in-plane bending vibrations—CH_2_
	Proline side chain interactions—in-plane bending vibrations—CH_2_
	Serine side chain interaction—in-plane bending vibrations—CH_2_
	Tryptophan side chain interaction—in-plane bending vibration—NH, stretching vibration—CC, in-plane bending vibration CH
	Tryptophan side chain interactions in-plane bending vibration—CH, stretching vibration—CC, CN
	Tyrosine side chain interactions in-plane bending vibrations—CH_2_
1399	Aspartic acid and glutamic acid—in-plane bending vibrations
	Aspartic acid side chain interaction—symmetric stretching—COO-, COH
	Glutamic acid side chain interactions—wagging vibrations—CH_2_
	Threonine—in-plane bending vibrations—COH, CH
	Tyrosine side chain interaction—wagging vibration—CH_2_
1233	Amide III
	Tyrosine side chain interactions—in-plane bending vibrations—COH
	Histidine interactions—in-plane bending vibrations CH, stretching vibrations—CN, and in-plane bending vibrations—NH
	Glutamic acid side chain interactions—stretching vibrations—C-O
	Glutamic acid side chain interactions—twisting vibrations—CH_2_, in-plane bending vibrations—CH
	Histidine side chain interactions—stretching vibrations—CN
	Threonine side chain interactions—in-plane bending vibrations—COH, CH
	Tryptophan side chain interactions—twisting vibrations—CH2, in-plane bending vibrations—CH
	Tyrosine—stretching vibration—CO, stretching vibrations—CC
859	CO_3_^2−^ bending vibrations

### 2.3. Swelling Ability of Biomaterials

**Figure 3:**
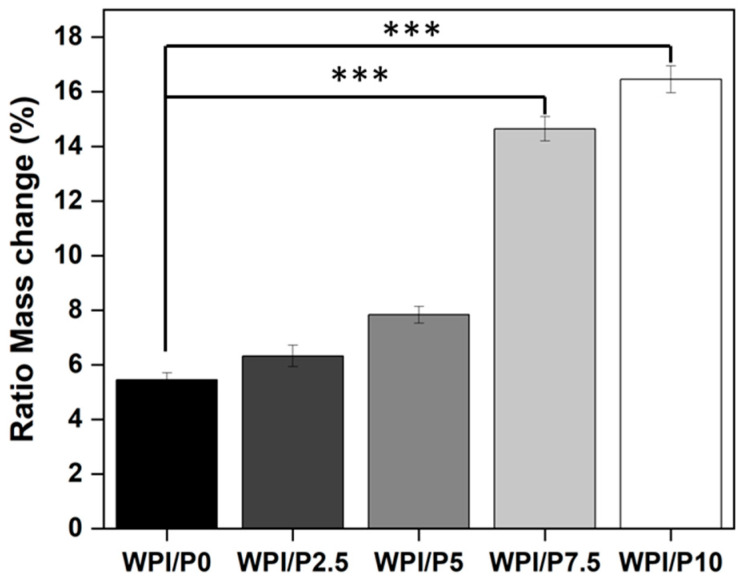
Results of swelling assays. The WPI/Pearl and WPI/P0 biomaterial samples were incubated at pH 7 for 5 days. The ratio mass change as a percentage was calculated. Each bar represents the mean ± SD of *n* = 10. *** *p* < 0.001–statistically significant results compared to the WPI/P0 base biomaterial (unpaired Student *t*-test, GraphPad Prism 10, Version 10.4.1 Software).

The ability to swell can be a desirable property of a scaffold due to an increase in pore size, due to stretching of the bonds, which provides an increase in internal surface area for cellular attachment. Additionally, the increasing solute uptake may conceivably favor cell nourishment. Increasing pearl concentration led to increasing swelling potential compared to the WPI/P0 base biomaterial (Figure 3). For instance, the swelling percentage increased from 5.45% for the WPI/P0 base biomaterial to 6.33% for WPI/P2.5., to 7.83% for the WPI/P5 group, to 14.64% for the WPI/P7.5 variable, and to 16.45% for the WPI/P10 sample groups. The WPI/P7.5 and the WPI/P10 demonstrated significant differences (*p* < 0.001) when compared to the WPI base biomaterial.

Whilst further investigation may be required to understand the molecular formation of the WPI/Pearl hydrogel, the observed trend could be attributed to the pearl particles preventing stronger cross-linking between the thiol bonds during the WPI gelation process. This is because increasing pearl powder concentration decreases protein content per unit volume. Additionally, in the process, the pearl acts as a physical barrier preventing denser cross-linking. The result is less elastic recovery and a more porous structure, and thus more swelling. Additionally, the addition of pearl increases the hydrophilicity, resulting in further solute uptake. Previously, both Norris et al. [51] and Ivory-Cousins et al. [37] demonstrated that the addition of inorganic material increases the swelling potential of the WPI hydrogel.

### 2.4. Mechanical Properties of Biomaterials

**Figure 4:**
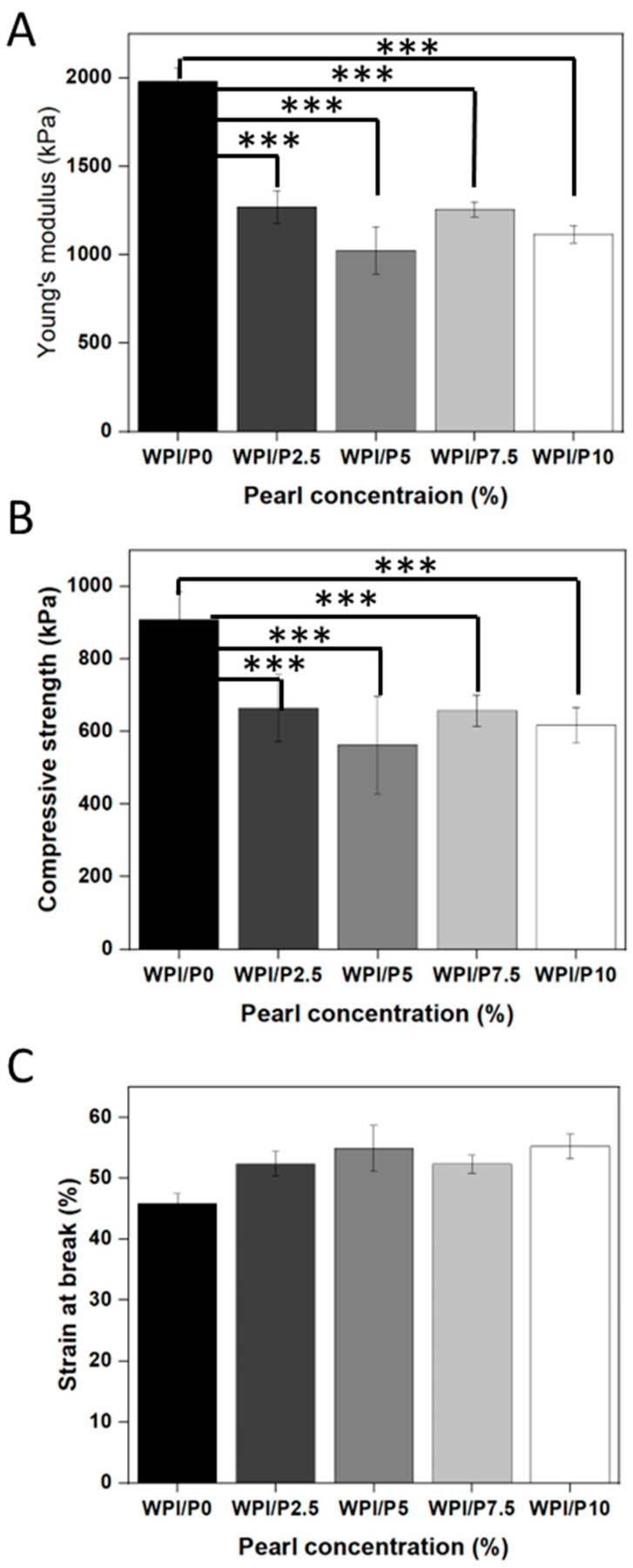
The results of the mechanical testing for the WPI/Pearl hydrogel. The Young’s modulus (**A**), the compressive strength (**B**), and the strain at break (**C**) were analyzed for each concentration sample group. Each bar represents the mean ± SD of *n* = 5. *** *p* < 0.001–statistically significant results compared to the WPI/P0 base biomaterial (unpaired Student *t*-test, GraphPad Prism 10, Version 10.4.1 Software).

Mechanical strength is a key attribute for tissue scaffolds. Therefore, the investigation analyzed the load-bearing potential of the WPI/Pearl hydrogel by analyzing the Young’s modulus, compressive strength, and the strain at break for all WPI/Pearl hydrogel variables. The result suggested that the addition of pearl to the WPI hydrogel significantly decreased (*p* < 0.001) the mechanical strength of the hydrogels (Figure 4A–C). For instance, the recorded Young’s modulus for the WPI/P0 base biomaterial was 1980 kPa, whereas the WPI/P10 variable demonstrated a Young’s modulus of 1117 kPa. Likewise, the compressive strength was reduced from 909 kPa for the base variable with no pearl to 617 kPa for WPI/P10, the maximum pearl concentration variable.

The reduction in mechanical strength could be attributed to the protein content per volume decrease in the hydrogel with increasing pearl powder concentrations. The cross-linking of the WPI hydrogel is formed mainly through interactions between cystine-containing thiol bonds and hydrophobic interactions. The addition of pearl powder inhibits bond formation, creating a weaker hydrogel with less dense cross-linking. Additionally, the interaction between pearl particles and proteins is weak, and the load is not transferred effectively, resulting in the pearl acting as a defect rather than a stabilizing molecule. This suggestion is further reinforced by the swelling analysis. For instance, the decrease in protein-to-protein bond formation could increase the swelling potential of the hydrogel whilst weakening mechanical properties.

Previously, the WPI hydrogel has demonstrated Young’s modulus values of approximately 2000 kPa [42,48,52]. The WPI/P0 base in this aligns with previous WPI mechanical analyses.

### 2.5. The Ability of Biomaterials to Undergo Proteolysis

**Figure 5:**
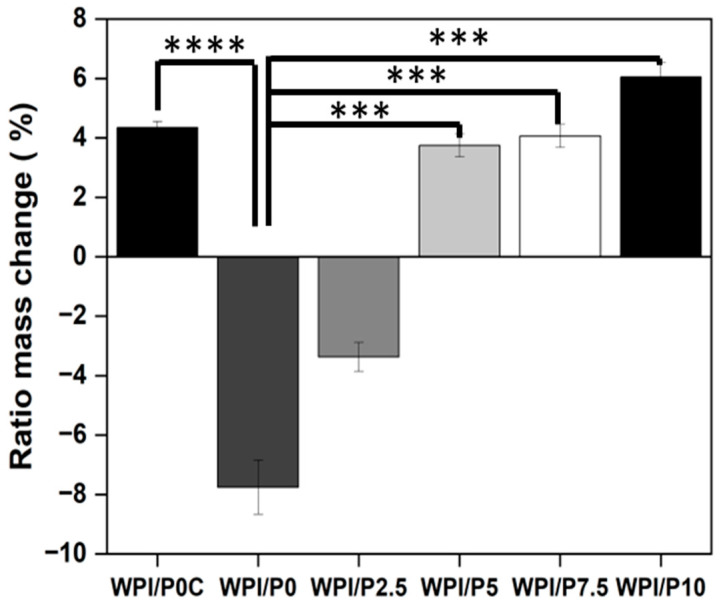
The results of the enzymatic degradation of the WPI/Pearl hydrogel samples. The samples were incubated at pH 7 with proteases for 5 days. The WPI0C sample group is a control without enzymes, whereas the WPI0 is the 40% WPI hydrogel control with enzymes in the solution. Each bar represents the mean ± SD of *n* = 10 (*** *p* < 0.001, **** *p* < 0.0001) compared to the WPI/P0C, one-way ANOVA, followed by Tukey’s multiple comparison test, GraphPad Prism 10, Version 10.4.1 Software).

The potential for a scaffold to undergo biodegradation is a key quality of a scaffold. Furthermore, the potential for predictive degradation is desirable. Therefore, an assay was undertaken to determine any effect a proteolytic enzyme would have on the degradation of the WPI/Pearl scaffolds. The results of the assay are observable in Figure 5. The result demonstrated that proteolytic enzymes aid in the degradation of the hydrogel, as demonstrated by a percentage mass increase of 4.34 for the WPI/P0C, the control with no added enzymes, compared to a percentage mass loss of −7.75 for the WPI/P0 control with added enzymes (*p* < 0.0001). It should be stated that the WPI hydrogel has been demonstrated to gain or lose mass in a pH 7 environment without an enzyme present. Likewise, previous investigations with protease have demonstrated a slight increase in mass, such as those previously demonstrated in [50,53]. This is because of the molecular interactions within the hydrogel network and the concentration of each gelation-causing mechanism. For example, at a neutral pH, the WPI protein side chains are charged and polar, creating a more hydrophilic hydrogel with the potential to attract and hold water and a hydrogel that can swell. Whereas mass loss can be caused by incomplete cross-linking or a decrease in the number of cross-linking agents, such as a reduction in the amino acid cystine.

The samples containing pearl powder lost mass when compared to the results from the swelling assay. However, the effect of the enzyme was less pronounced as the pearl concentration increased, compared to the WPI/P0 samples (*p* < 0.001). For instance, the WPI/P2.5 variable demonstrated a mass increase of −3.37, the WPI/P5 variable a 3.74 percentage mass increase, the WPI/P7.5 sample a 4.06 percentage mass increase, and the WPI/P10 variable demonstrated a 6.04 percentage mass increase. This suggests that the addition of pearl can reduce the rate of degradation, especially when considering the increase in mass with an increasing pearl concentration, when comparing the pearl-containing sample to the WPI/P0 control. The addition of pearl into the hydrogel decreases the volume of accessible, cleavable bonds. Additionally, being a mineral phase, the pearl is non-degradable by proteolytic enzymes. The decrease in degradation rate with an increasing pearl concentration could potentially result in predictive degradation.

### 2.6. The Ability of Biomaterials to Release Calcium Ions

**Figure 6:**
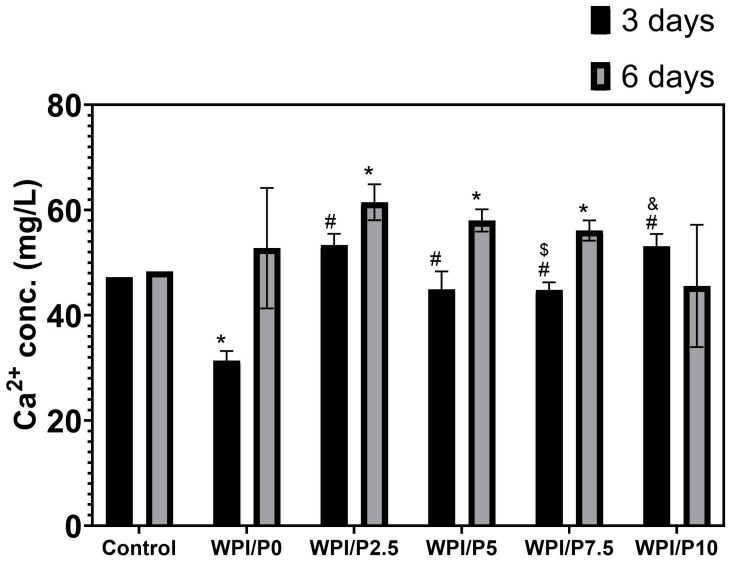
The ability of biomaterials to absorb or release calcium ions over time. During the experiment, liquid extracts from biomaterials (*n* = 4) were harvested on day 3, and then a new portion of culture medium was added. The extracts were taken once again on day 6. *-Statistically significant difference compared to the control extract–culture medium incubated without biomaterials and collected at the specified time point; #-statistically significant difference compared to the extract from base biomaterial (WPI/P0), collected at the specified time point; $-statistically significant difference to the extract from WPI/P2.5 biomaterial, collected at the specified time point; &-statistically significant difference compared to the extract from WPI/P7.5 biomaterial, collected at the specified time point. Two-way ANOVA, followed by Bonferroni comparison test, *p* < 0.05, GraphPad Prism 10, Version 10.4.1 Software.

This experiment showed (Figure 6) that after 3 days of incubation, the base biomaterial (WPI/P0) displayed a significant ability (*p* < 0.05) to absorb calcium ions from the culture medium–the concentration of these ions was approximately 1.5 times lower than in the culture medium (marked as control). For all other biomaterials, i.e., those containing pearl powder, the concentration of calcium ions in the extracts obtained after 3 days of incubation was comparable to the control or slightly higher, which may indicate that the addition of pearl powder inhibits the absorption properties of the biomaterial composed solely of WPI, or may suggest that a compensatory effect occurs, i.e., the WPI matrix has absorption properties, while the pearl powder contained in the biomaterials dissolves and releases calcium ions into the aqueous environment, equalizing their concentration. In turn, on the 6th day of the experiment (Figure 6), the concentration of calcium ions in the extract from the base biomaterial was observed to be comparable to that obtained for the culture medium incubated without biomaterials (control), which may indicate that this biomaterial exhibits calcium ion absorption properties only during the first few days. In turn, in the case of most biomaterials containing pearl powder (WPI/P2.5, WPI/P5, and WPI/P7.5), it was noted that the concentration of calcium ions in the obtained extracts was significantly higher (*p* < 0.05), compared to the level of these ions in the culture medium (control). These results may therefore confirm that after a few days of incubation, the absorption effect caused by the WPI matrix ceases, while calcium ions are still released by the dissolving pearl powder. Interestingly, in the case of the biomaterial containing the highest amount of pearl powder (WPI/P10), the results were not significantly higher, but rather comparable to the control. This result, however, may result from a relatively high standard deviation obtained for the samples. This experiment was carried out to gain knowledge about the ability of biomaterials to absorb or release calcium ions. It is motivated by the fact that calcium ions play a very important role in bone metabolism, among others, acting as a building material for bone and playing a key role in bone remodeling and cell signaling. They also support the growth, proliferation, and differentiation of bone-forming cells—osteoblasts [54,55,56,57,58]. Therefore, it seems that biomaterials containing pearl powder should have a positive effect on osteoblast response.

### 2.7. The Ability of Biomaterials to Release Proteins

**Figure 7:**
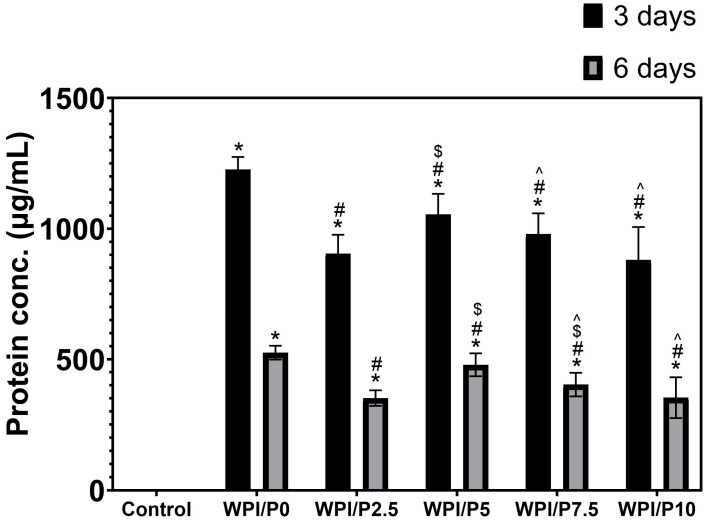
The ability of biomaterials to release protein over time. During the experiment, liquid extracts from biomaterials (*n* = 4) were harvested on day 3, and then a new portion of culture medium was added. The extracts were taken once again on day 6. *-Statistically significant difference compared to the control extract–culture medium incubated without biomaterials and collected at the specified time point; #-statistically significant difference compared to the extract from base biomaterial (WPI/P0), collected at the specified time point; $-statistically significant difference to the extract from WPI/P2.5 biomaterial, collected at the specified time point; ^-statistically significant difference compared to the extract from WPI/P5 biomaterial, collected at the specified time point. Two-way ANOVA, followed by Bonferroni comparison test, *p* < 0.05, GraphPad Prism 10, Version 10.4.1 Software).

This experiment revealed (Figure 7), first and foremost, that all tested biomaterials (both the base and those containing pearl powder) had a significant (*p* < 0.05) ability to release protein into the culture medium (marked as control). Furthermore, the biomaterials were shown to have the highest protein release capacity within the first three days. This ability was, importantly, lower in the following days of the experiment. Furthermore, all tested biomaterials containing pearl powder released significantly lower amounts of protein (approx. 1.1×–1.4×) compared to the base biomaterial on both days 3 and 6 of the experiment. This is likely due to the biomaterial composition. The extracts from biomaterials were prepared at a ratio of 0.1 g of scaffold per 1 mL of culture medium; hence, the biomaterials containing pearl powder contained less WPI than the base biomaterial consisting solely of WPI. The ability of biomaterials to release protein is an important parameter for predicting cellular response. Previous studies conducted by our research group have clearly shown that WPI-based biomaterials that release this protein into the culture medium support osteoblast growth, proliferation, and differentiation [59,60,61].

### 2.8. Cell Culture Experiments In Vitro

#### 2.8.1. Cell Viability in Indirect Contact

**Figure 8:**
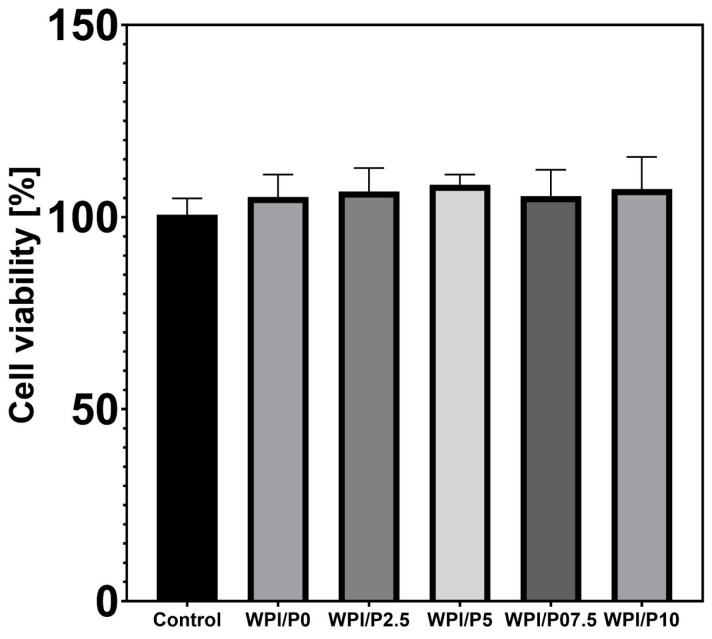
Viability of normal human osteoblasts treated with biomaterial extracts (*n* = 4) for 24 h. Cell viability was determined by the MTT assay. No statistically significant differences were observed between groups (*p* > 0.05, based on one-way ANOVA, followed by Tukey’s multiple comparison test, *p* < 0.05, GraphPad Prism 10, Version 10.4.1 Software).

The MTT assay demonstrated (Figure 8) that extracts obtained from all tested biomaterials did not exhibit cytotoxic effects in vitro against normal human osteoblasts. Although increased viability was observed in cells treated with biomaterial extracts (up to approx. 105–108%) compared to the viability of cells incubated with the control extract, 100% (culture medium incubated without biomaterials), the obtained results were not statistically significant (*p* > 0.05). The observed enhanced cell viability after treatment with biomaterial extracts is most likely related to the ability of scaffolds to release protein and calcium ions into the culture medium (see Section 2.6 and Section 2.7).

#### 2.8.2. Cell Viability in Direct Contact

**Figure 9:**
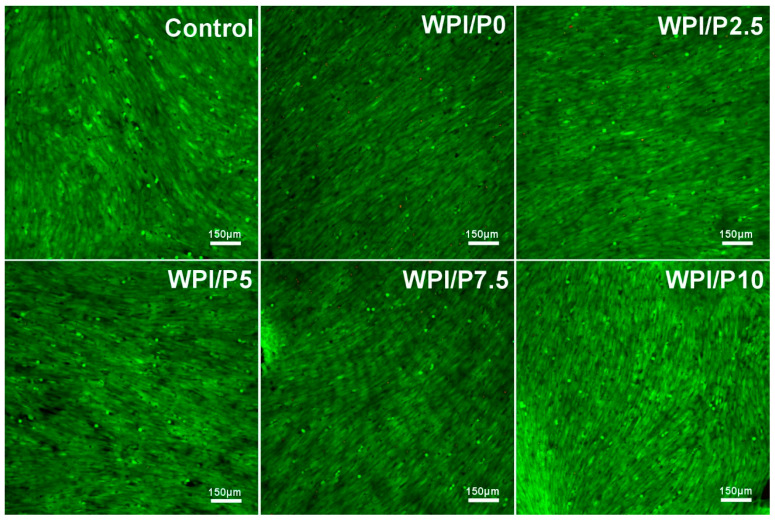
Viability of normal human osteoblasts growing on control biomaterials–polystyrene and tested biomaterials composed of WPI without or with pearl powder (WPI/P0 and WPI/P2.5; WPI/P5; WPI/P7.5; WPI/P10, respectively). After 48 h of incubation, cells were differentially stained using the Live/Dead Double Cell Staining Kit and observed using a confocal laser scanning microscope (CLSM). Only living cells were observed, which emitted green fluorescence (if dead cells were present, they would emit red fluorescence). Magnification = 100×, bar scale = 150 μm.

Differential staining of live and dead cells (Figure 9) demonstrated that the surfaces of all tested biomaterials supported osteoblast colonization and growth. Similar to cells cultured on polystyrene (control), cells cultivated on biomaterials exhibited very high viability, as evidenced by visible green fluorescence. Hence, these observations showed that the surfaces of the fabricated biomaterials were safe and beneficial for cell growth. It should be emphasized that these results were expected, as both WPI-based biomaterials and pearl powder have the ability to enhance cell growth [23,36,37,61].

#### 2.8.3. Cell Proliferation in Direct Contact

**Figure 10:**
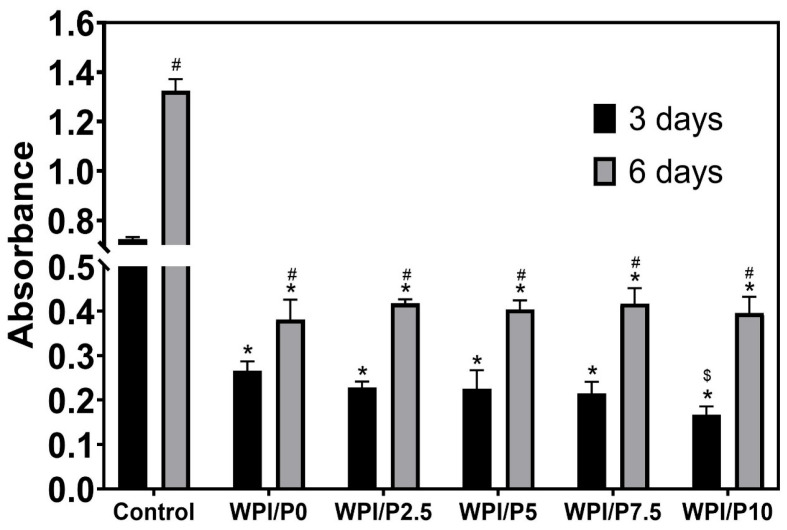
Quantitative assessment of proliferation of normal human osteoblasts cultured on control biomaterials–polystyrene (*n* = 4) and tested biomaterials (*n* = 4) composed of WPI without or with pearl powder (WPI/P0 and WPI/P2.5; WPI/P5; WPI/P7.5; WPI/P10, respectively). After 3 and 6 days of incubation, cell metabolic activity was assessed using the WST-8 assay. The OD value is directly proportional to the number of viable, metabolically active cells. *–Statistically significant difference compared to the control biomaterial–polystyrene at the specified time point; #–statistically significant difference compared to the results obtained after 3 and 6 days of incubation; $-statistically significant difference between WPI/P0 and WPI/P10 biomaterial on day 3. Two-way ANOVA, followed by Bonferroni comparison test, *p* < 0.05, GraphPad Prism 10, Version 10.4.1 Software).

**Figure 11:**
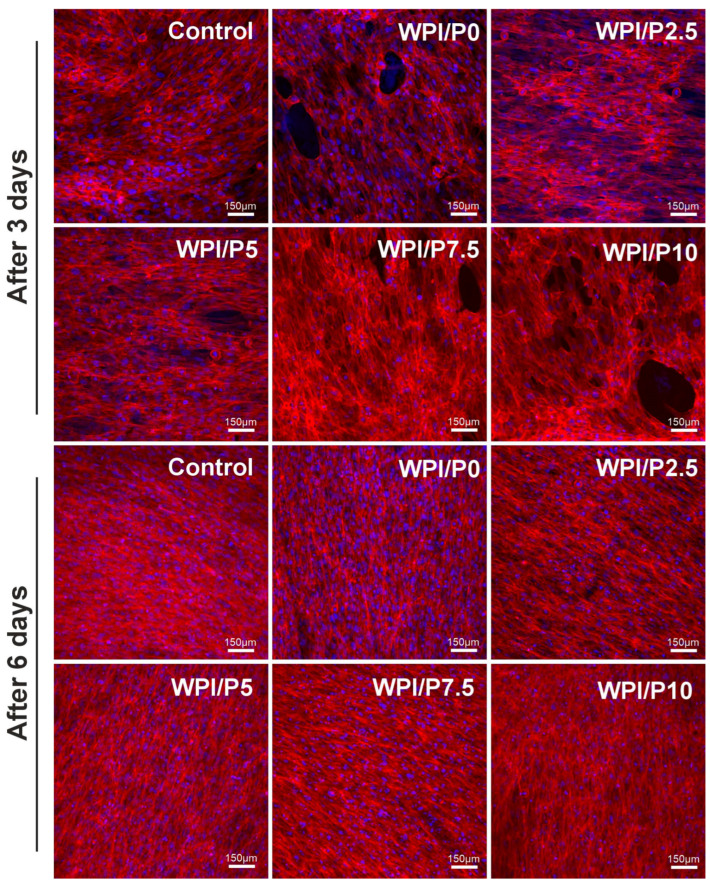
Qualitative assessment of proliferation of normal human osteoblasts cultured on control biomaterials–polystyrene and tested biomaterials composed of WPI without or with pearl powder (WPI/P0 and WPI/P2.5; WPI/P5; WPI/P7.5; WPI/P10, respectively). After 3 and 6 days of incubation, cells were stained using the Hoechst 33342 and AlexaFluor^TM^ 635 Phalloidin and observed using a confocal laser scanning microscope (CLSM). Live cells = green fluorescence; dead cells = red fluorescence. Magnification = 200×, bar scale = 150 μm.

The WST-8 test (Figure 10) demonstrated that all tested biomaterials (control and WPI-based) promoted cell proliferation over time, as the OD values were higher with increasing incubation time. Unfortunately, no significant differences (*p* > 0.05) were found between biomaterials containing WPI and pearl powder, which was somewhat surprising, as a higher powder content was expected to favorably influence cell division. Microscopic observations (Figure 11) performed after fluorescent staining of cell nuclei and actin filaments of the cytoskeleton showed that the surfaces of all biomaterials were favorable for osteoblast division over time. At the end of the experiment (on day 6), a monolayer of cells with normal morphology was observed. Therefore, this experiment demonstrated that all tested biomaterials similarly promoted osteoblast division over time. According to the literature data, both WPI and pearl powder have the ability to enhance cell proliferation [19,28,48]. Perhaps it would be worth considering increasing the pearl powder content in WPI-based biomaterials to observe an enhanced effect.

#### 2.8.4. Cell Differentiation in Direct Contact

**Figure 12:**
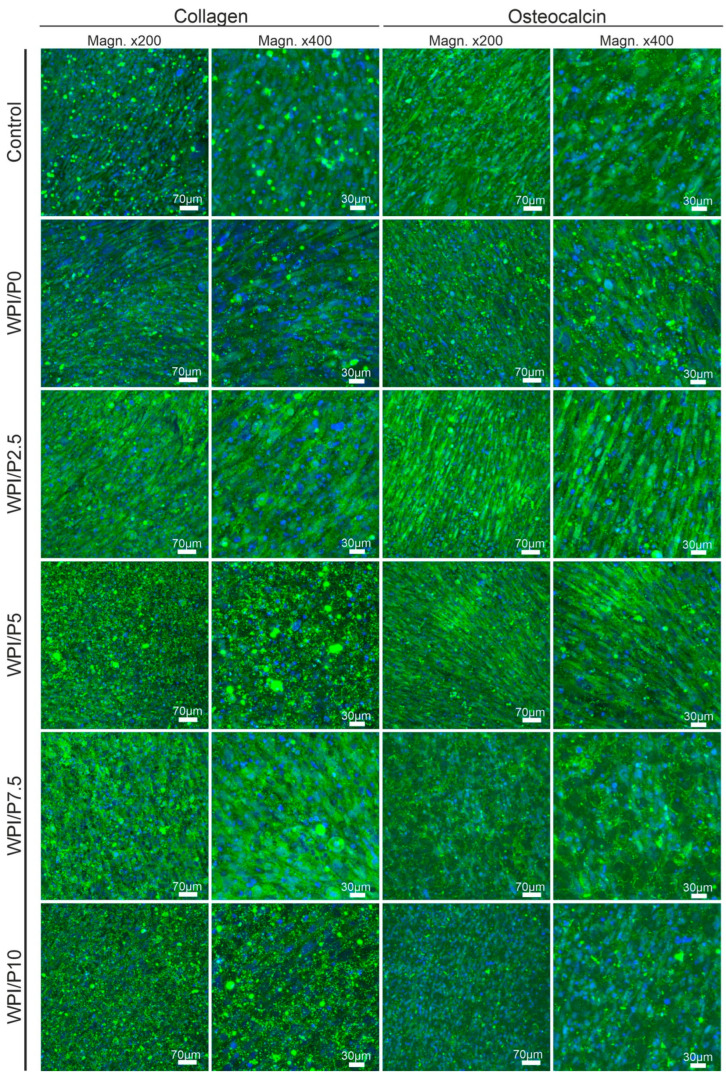
Qualitative assessment of osteogenic differentiation of normal human osteoblasts cultured on control biomaterials–polystyrene and tested biomaterials composed of WPI without or with pearl powder (WPI/P0 and WPI/P2.5; WPI/P5; WPI/P7.5; WPI/P10, respectively). After 21 days of incubation, cells were incubated with primary anti-collagen I antibody or primary anti-osteocalcin antibody, followed by staining with specified secondary antibody-conjugated with Alexa Fluor 488 and additionally with Hoechst 33342. Then, the cells were observed using a confocal laser scanning microscope (CLSM). Cell nuclei = blue fluorescence; collagen or osteocalcin = green fluorescence. Magnification = 200× or 400×, bar scale = 70 or 30 μm.

Microscopic observations (Figure 12) performed after 21 days of incubation showed that osteoblasts cultured on the control biomaterial (polystyrene) and on scaffolds containing WPI (basic and with added pearl powder) demonstrated normal osteogenic differentiation capacity, as the cells demonstrated the ability to synthesize two key markers: type I collagen and osteocalcin. Therefore, this experiment demonstrated that the surfaces of all tested biomaterials are highly conducive to osteogenic differentiation of human osteoblasts. It is known that both biomaterials containing WPI and pearl powder have the ability to support cell differentiation [25,37,61,62].

### 2.9. In Vivo Toxicological Assessment Using Zebrafish Larvae (Danio Rerio)–FET Test

**Figure 13:**
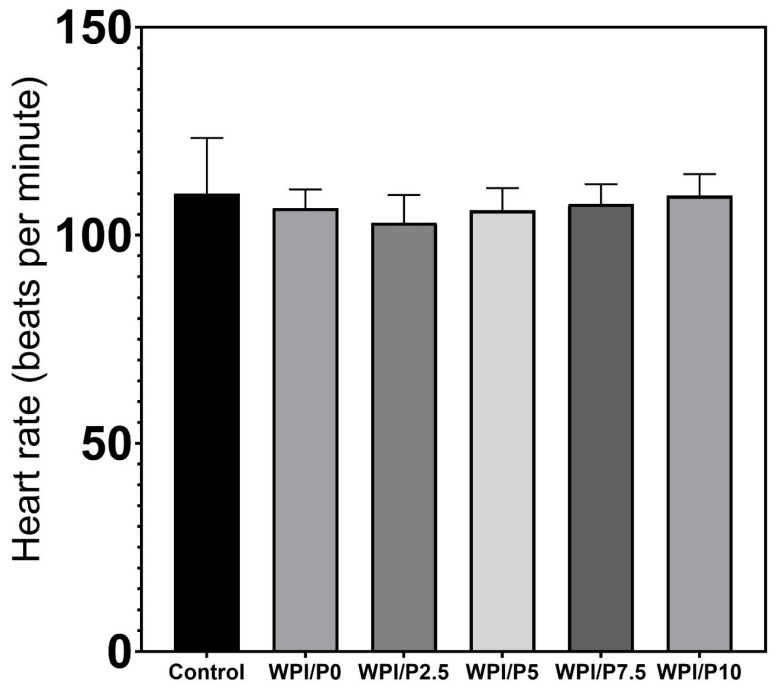
Heart rate at 96 hpf in the FET test. Heart rate was measured manually for exactly 10 s and multiplied by 6, results presented as mean with SD, *n* = 12. The results were not statistically significant among themselves, based on one-way ANOVA, followed by Tukey’s multiple comparison test, *p* < 0.05, GraphPad Prism 10, Version 10.4.1 Software).

**Figure 14:**
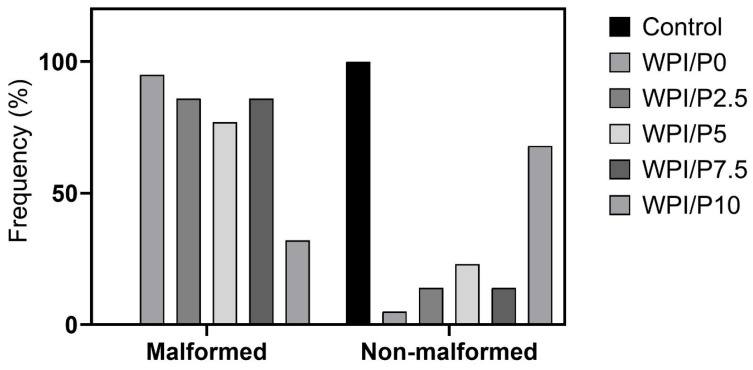
Distribution of Malformed vs. Non-Malformed larvae. Malformation was defined as pericardial oedema.

**Figure 15:**
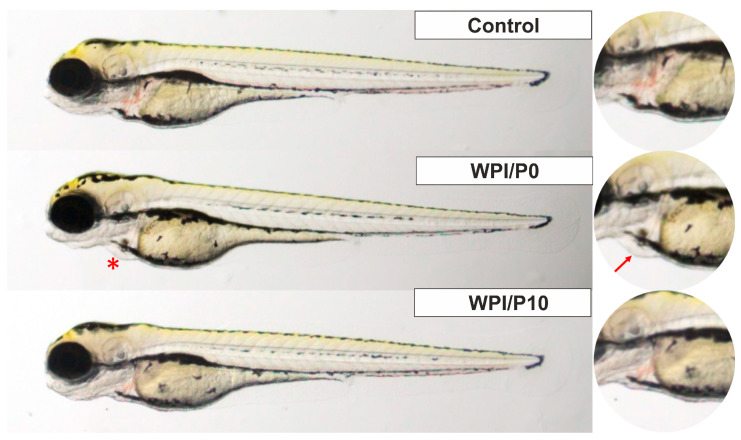
Zebrafish larvae in lateral position at 96 hpf of the FET test. A red asterisk (*) indicates the presence of pericardial oedema, also shown in the right panel with a red arrow. Images were taken using a SteREO Discovery.V8 microscope (Carl Zeiss Microscopy GmbH, Hallbergmoos, Germany) at 3.2× magnification.

**Figure 16:**
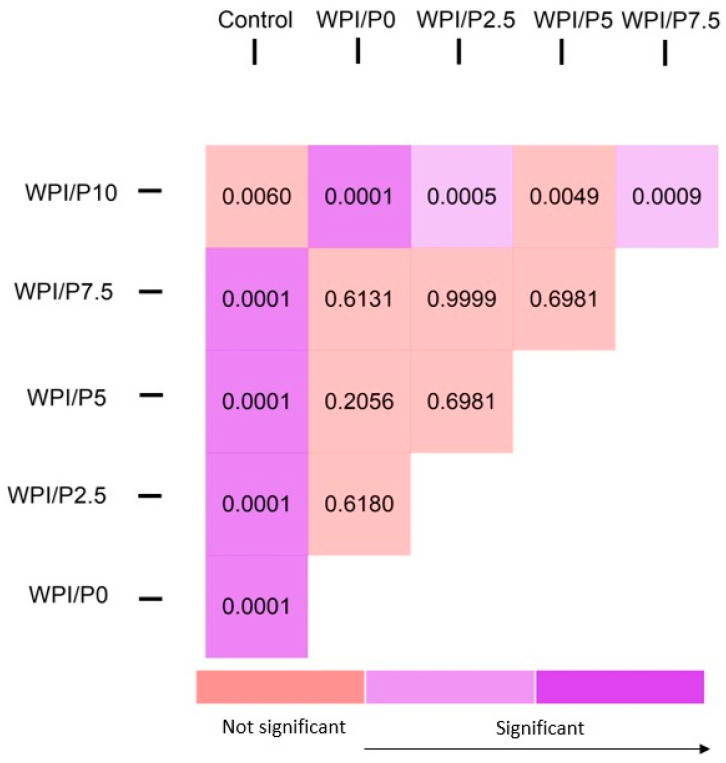
Heatmap of Bonferroni-corrected *p*-values from Fisher’s exact test (2 × 2) for all group pairs.

Three mortalities due to coagulation were recorded during the experiment, with one occurring in each of the control, WPI/P0, and WPI/P7.5 groups, resulting in a survival rate of 95.83% (Table 2). This falls within the acceptable limits of the OECD Guideline, and the tested extracts can be considered as non-toxic under the applied experimental conditions. Moreover, no abnormalities in larval hatching were observed, and each group achieved the required hatching rate of over 80% at 96 hpf (Table 1). Also, the Kruskal–Wallis test did not reveal any differences in heart rate between the control and biomaterial groups [H(5) = 6.304, *p* = 0.2778] (Figure 13). However, during the morphological evaluation, we observed a consistent abnormality from the physiological state, manifested as the presence of pericardial oedema, which could be classified as mild (Figure 14). The incidence of this pathology was high in the WPI/P0, WPI/P2.5, WPI/P5, and WPI/P7.5 groups, amounting to 94.12%, 86.36%, 77.27%, and 85.71%, respectively, and was notably lower in the WPI/P10 group, where it reached 31.58% (Figure 15). To see if malformation rates differ significantly, we used Fisher’s exact test (2 × 2) with Bonferroni correction (α’ = 0.05/15 = 0.0033). In all groups except the WPI/P10 group, the incidence of malformations was significantly higher compared to the control group and did not differ significantly between those experimental groups. The WPI/P10 group was the only one in which the frequency of malformations did not differ significantly from the control and was markedly lower than in the other groups (Figure 16). Importantly, as noted previously, this alteration did not impact heart rate, survival, and hatching rate.

## 3. Materials and Methods

### 3.1. Fabrication of Biomaterials

**Figure 17:**
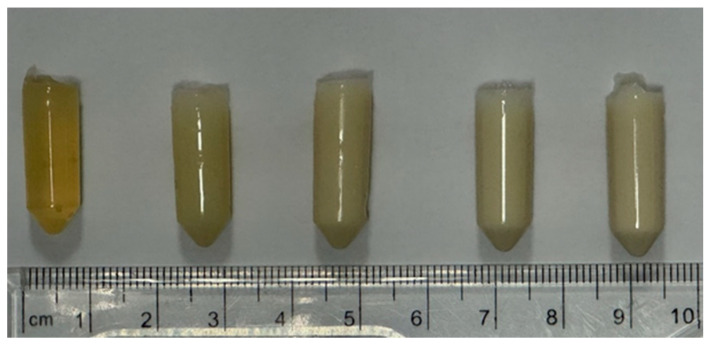
An image of the WPI/Pearl hydrogels post-sterilization. From left to right–WPI/P0, WPI/P2.5, WPI/P5, WPI/P7.5 and WPI/P10.

WPI/Pearl hydrogels were formed from WPI sourced from Davis and Co., North Ridgeville, Ohio, USA. This investigation utilized 40% WPI hydrogel (*w/v*) with an additional 2.5%, 5%, 7.5%, and 10% of pearl powder, as per Table 3. The solutions were homogenized for 24 h at 20 rpm on an IKA Loopster (IKA England LTD, Oxford, UK). Post-homogenization, the samples were degassed and formed into 1 mL/1 g samples for analysis. Gelation was achieved at 70 °C for 5 min. Sterilization was achieved via autoclaving. The formed hydrogel post-sterilization can be observed in Figure 17.

### 3.2. Scanning Electron Microscopy and Stereoscopic Microscopy

Scanning electron microscopy (SEM) was used to analyze the effect on hydrogel formation with the addition of pearl and to evidence the incorporation of pearl into the hydrogel. The assay was conducted with a JSM-6390 LV, JEOL Ltd. (Welwyn Gardens, UK), scanning electron microscope with an accelerating voltage of 15 kV. Dehydrated samples from the center of the hydrogel were gold-coated and imaged at ×3000 magnification. In turn, a stereoscopic microscope (Olympus SZ61TR, Olympus, Warszawa, Poland) was utilized in order to visualize the macrostructure of biomaterials.

### 3.3. Evaluation of Chemical Interactions by FTIR

FTIR was utilized to determine the successful incorporation of pearl into the hydrogel. WPI/Pearl hydrogel samples were cut to a thickness of 0.5 mm. The samples were dehydrated. Post-dehydration, the spectra were analyzed with a Cary 630 FTIR spectrophotometer (Agilent, Santa Clara, CA, USA). The investigation analyzed a spectral range of between 650 and 4000 cm^−1^, with 32 scans per sample.

### 3.4. Evaluation of Water Uptake

Swelling analysis was conducted to determine the effect of the incorporation of pearl powder on the swelling potential of the WPI hydrogels in a neutral environment. The method utilized was as follows: WPI/Pearl hydrogel samples with a mass of 1 g were placed in 5 mL and incubated for 5 days at 37 °C in PBS. Post-incubation, the ratio mass change was calculated as a percentage, where the swelling percentage (S%) was calculated from the wet mass (Mw) and the dry mass (Md).

S% = (Mw − Md)/Md × 100

### 3.5. Evaluation of Young’s Modulus and Compressive Strength

Mechanical analysis was conducted on the WPI/Pearl hydrogel variables. The samples were formed with a height of 10 mm and a radius of 4 mm. The mechanical compressive analysis utilized an Instron 3345 (Instron, High Wycombe, UK). The rate of compression was 2 mm/min. The Young’s modulus, compressive strength, and strain at the break of the hydrogels were calculated using the following formulae.

Young’s modulus (Ε) was calculated as follows:

Ε = σ/∈

where σ denotes stress, and ε denotes strain.

Compressive strength was calculated as follows:

F = P/(πr2)

where P is the load at failure, and A is the cross-sectional area.

Strain at break was calculated as follows:

∈ = ∆L/L × 100

where ΔL is the difference between the initial length and the final length, and L is the initial length.

### 3.6. Evaluation of Proteolysis

The use of WPI/Pearl hydrogel as a scaffold introduces the hydrogel to proteolytic enzymes. Therefore, an assay was conducted to determine the effects of enzymes on the hydrogel. The investigation utilized proteases from Sigma (ThermoFisher Loughborough, Loughborough, UK), compared to known concentrations of collagenase levels, adapted from [63]. WPI/Pearl hydrogels with concentrations consistent with the investigation, with a mass of 1 g, were placed in a 5 mL PBS/collagenase solution. The samples were incubated at 37 °C for 5 days. The percentage ratio mass change was calculated, where the swelling percentage (S%) was calculated from the wet mass (Mw) and the dry mass (Md).

S% = (Mw − Md)/Md × 100

### 3.7. Evaluation of Calcium Ions Profile

Before the experiment, liquid extracts from the biomaterials were prepared according to the ISO 10093-12:2012 standard [64]. For this purpose, the biomaterials were placed in multiwell plates and covered with culture medium (0.1 g of biomaterial per 1 mL of liquid). Culture medium without biomaterials served as the experimental control. After 3 days of incubation at 37 °C, the extracts were collected, and a new portion of medium was added to the wells. Extraction was continued for another 3 days at 37 °C (total experiment duration: 6 days). Calcium ion concentration was determined using a commercial assay–Calcium CPC ion detection kit according to the manufacturer’s protocol (Biomaxima, Lublin, Poland).

### 3.8. Evaluation of Protein Profile

This experiment was performed analogously to the calcium ion concentration measurement described in detail in Section 3.6. For evaluation of calcium ion profile, the concentration of released protein was determined using a commercial assay–Pierce^TM^ BCA Protein Assay Kit according to the manufacturer’s recommendations (ThermoFisher Scientific, Waltham, MA, USA).

### 3.9. Evaluation of Influence of Biomaterial’s Extract on Osteoblast Viability

Osteoblast (hFOB 1.19, ATCC, Manassas, VA, USA) viability was assessed based on ISO 10093-5:2009 guidelines [65]. Liquid extracts from biomaterials were prepared according to ISO 10093-12:2012 [64], at a ratio of 0.1 g per ml of culture fluid, i.e., DMEM/F12 1:1 medium (Gibco ThermoFisher Scientific, Waltham, MA, USA), supplemented with penicillin (10.000 units)–streptomycin (10 mg/mL) solution (Merck, Warsaw, Poland), and 10% fetal bovine serum (FBS, Pan-Biotech, Aidenbach, Germany). Extraction was carried out for 24 h at 37 °C. The hFOB 1.19 cells were seeded into 96-well plates (100 µL/well) at a density of 2 × 10^4^ cells/well in culture medium. The collected extracts were then added to osteoblasts and incubated for 24 h. Cell viability was determined using the MTT assay. More details about this procedure and cell maintenance are described in our previous publications [61,66].

### 3.10. Evaluation of Influence of Biomaterial’s Surface on Osteoblast Viability

The viability of osteoblasts (hFOB 1.19, ATCC, Manassas, VA, USA) cultured directly on biomaterials was assessed after 48 h of incubation. Briefly, cells (500 µL) were seeded at a density of 2 × 10^4^ cells/disc directly onto biomaterials arranged in a 48-well plate or into empty plate wells (PS, control). After incubation, cells were stained using the Live/Dead Cell Double Staining Kit (Merck, Warsaw, Poland) and observed under CLSM. More details about this procedure and cell maintenance are described in our previous publications [38,66].

### 3.11. Evaluation of Influence of Biomaterial’s Surface on Osteoblast Proliferation

The proliferation of osteoblasts (hFOB 1.19, ATCC, Manassas, VA, USA) cultured directly on biomaterials was assessed after 3 and 6 days of incubation. Briefly, cells (500 µL) were seeded at a density of 1 × 10^4^ cells/disc directly onto biomaterials arranged in a 48-well plate or into empty plate wells (PS, control). After incubation, metabolic activity was assessed using the WST-8 assay (Merck, Warsaw, Poland). Then, cells were stained using Hoechst 33342 (Merck, Warsaw, Poland) dye and AlexaFluor^TM^ 635 Phalloidin (ThermoFisher Scientific, Waltham, MA, USA) dye and observed under CLSM. More details about this procedure and cell maintenance are described in our previous publications [38,61,66].

### 3.12. Evaluation of Influence of Biomaterial’s Surface on Osteoblast Differentiation

The osteogenic differentiation of osteoblasts (hFOB 1.19, ATCC, Manassas, VA, USA) cultured directly on biomaterials was assessed after 21 days of incubation. Briefly, cells (500 µL) were seeded at a density of 2 × 10^4^ cells/disc directly onto biomaterials arranged in a 48-well plate or into empty plate wells (PS, control). After incubation, the cells were stained with primary rabbit/IgG polyclonal anti-collagen I antibody (Invitrogen, ThermoFisher Scientific, Waltham, MA, USA), diluted 1:100 in 0.1% bovine serum albumin, BSA (Merck, Warsaw, Poland), or primary rabbit/IgG polyclonal anti-osteocalcin antibody (Bioss, ThermoFisher Scientific, Waltham, MA, USA), diluted 1:100 in 0.1% BSA (Merck, Warsaw, Poland), followed by staining with secondary goat polyclonal anti-rabbit IgG (H + L) antibody-conjugated with AlexaFluor^®^488 (Abcam, Cambridge, UK)**,** and diluted 1:1000 in PBS (Merck, Warsaw, Poland). Hoechst 33342 (Merck, Warsaw, Poland) was used to visualize cell nuclei. The cells were observed under CLSM. More details about this procedure and cell maintenance are described in our previous publication [61].

### 3.13. Evaluation of Influence of Biomaterial’s Extract on Toxicity In Vivo

This experiment was conducted using zebrafish larvae (AB strain) in accordance with OECD Test Guideline No. 236. Within four hours post-fertilization (hpf), zebrafish embryos (12 per group × 2 replicates, total *n* = 24) were transferred into 96-well plates and incubated with 150 µL of either control solution (E3 medium) or biomaterial extracts (they were prepared as described previously in Section 3.6). Incubation was carried out at 28.5 °C under a 14/10 h light/dark cycle (Incubator IN110, Memmert GmbH + Co., Schwabach, Germany). The test solutions were refreshed every 24 h. Prior to each solution change, four apical endpoints were assessed as indicators of lethality: (i) coagulation, (ii) absence of somite formation, (iii) failure of tail detachment from the yolk sac, and (iv) absence of heartbeat (OECD No 236) [67]. After 96 h, larvae were immobilized using 1.5% methylcellulose, and the heart rate was manually measured over a 10 s interval and multiplied by 6 (*n* = 12). Subsequently, larvae were anesthetized with tricaine, and any malformations were documented using a SteREO Discovery.V8 stereomicroscope (Zeiss, Jena, Germany). Additionally, the hatching rate was recorded at 96 h hpf. Following all assessments, larvae were euthanized using a tricaine overdose (300–500 µg/mL) [38,68].

## 4. Conclusions

In this work, we fabricated biomaterials containing WPI and pearl powder and assessed their potential as future bone scaffolds. Of the evaluated variants, the biomaterial containing the highest pearl powder content, i.e., 10% *w/v* (termed WPI/P10), demonstrated the best properties. This biomaterial possessed a porous structure and high absorption properties, allowing it to be impregnated with bioactive substances and preventing proteolysis. Unfortunately, despite the highest pearl powder content, this biomaterial did not demonstrate improved mechanical properties or increased calcium ion release capacity. In vitro cell culture studies showed that this biomaterial not only exhibits no cytotoxicity but also promotes the growth, proliferation, and osteogenic differentiation of normal human osteoblasts. Furthermore, studies using the Danio rerio model demonstrated that this biomaterial does not cause toxic effects or any malformations. Therefore, the WPI/P10 biomaterial appears to be a promising bone scaffold option. However, the in vivo studies performed have certain limitations, and additional in vivo studies, including implantation procedures in animals, are necessary to assess the behavior of the biomaterial after implantation. Hence, we plan to conduct further studies in the future, including attempts to increase the pearl powder content in WPI-based biomaterials and expanding the in vivo testing panel, including an implantation procedure in rodents.

## Figures and Tables

**Table 2:** Survival and hatching rate (%) at 96 hpf in the FET test.

	Control	WPI/P0	WPI/P2.5	WPI/P5	WPI/P7.5	WPI/P10.0
Survival	95.83	95.83	100	100	95.83	100
Hatching	100	100	95.83	100	95.65	95.83

**Table 3:** The WPI/Pearl hydrogel samples and their constituent concentrations.

	% WPI	% Pearl Powder
WPI/P0	40	0
WPI/P2.5	40	2.5
WPI/P5	40	5
WPI/P7.5	40	7.5
WPI/P10	40	10

## Data Availability

Data are available on reasonable request.

## Abbreviations

The following abbreviations are used in this manuscript:

ACP	amorphous calcium phosphate
BMPs	bone morphogenetic proteins
BSA	bovine serum albumin
CaCO_3_	calcium carbonate
CaSiO_3_	calcium silicate
CLSM	confocal laser scanning microscope
CO_2_	carbon dioxide
CSDs	critical-sized defects
FTIR	Fourier-Transform Infrared Spectroscopy
FBS	fetal bovine serum
GelMa	gelatin methacrylate
Hap	Hydroxyapatite
PLA	polylactic acid
SEM	scanning electron microscopy
TCP	tricalcium phosphate
TEP	tissue engineering products
TTCP	tetracalcium phosphate
VEGF	vascular endothelial growth factor
WPI	whey protein isolate
